# Nectin-4 as Blood-Based Biomarker Enables Detection of Early Ovarian Cancer Stages

**DOI:** 10.3390/cancers14235867

**Published:** 2022-11-29

**Authors:** Christoph Rogmans, Julia Feuerborn, Leonie Treeck, Nils Tribian, Inken Flörkemeier, Norbert Arnold, Jörg Paul Weimer, Nicolai Maass, Peer Jansen, Wolfgang Lieb, Astrid Dempfle, Dirk O. Bauerschlag, Nina Hedemann

**Affiliations:** 1 Department of Gynecology and Obstetrics, Christian-Albrechts-University Kiel and University Medical Center Schleswig-Holstein Campus Kiel, 24105 Kiel, Germany; 2 Department of Medicine II, Hematology and Oncology, Christian-Albrechts-University Kiel and University Medical Center Schleswig-Holstein Campus Kiel, 24105 Kiel, Germany; 3 Institute of Epidemiology, Christian-Albrechts-University Kiel and University Medical Center Schleswig-Holstein Campus Kiel, 24105 Kiel, Germany; 4 Institute of Medical Informatics and Statistics, Kiel University and University Medical Center Schleswig-Holstein Campus Kiel, 24105 Kiel, Germany

**Keywords:** ovarian cancer, Nectin-4, HB-EGF, Amphiregulin, tumor marker, early detection, liquid biopsy

## Abstract

**Simple Summary:**

Ovarian cancer is usually diagnosed in an advanced stage of the disease and is associated with a poor prognosis. Therefore, it is important to find reliable biomarkers that allow early diagnosis. In this study, we detected elevated Nectin-4 and HB-EGF serum levels in ovarian cancer patients which correlate with prognostically favorable parameters, such as early FIGO stage and successful tumor debulking. In addition, Nectin-4 was elevated in patients with low blood levels of Ca-125. This suggests that Nectin-4 and Ca-125 provide complementary diagnostic information. Therefore, Nectin-4 and HB-EGF appear to be promising blood-based biomarkers for the diagnosis of early stages of ovarian cancer.

**Abstract:**

Ovarian cancer is the third most common gynecological malignancy and has the highest mortality rate. Owing to unspecific symptoms, ovarian cancer is not detected until an advanced stage in about two-thirds of cases. Therefore, it is crucial to establish reliable biomarkers for the early stages to improve the patients’ prognosis. The aim of this study is to investigate whether the ADAM17 substrates Nectin-4, Heparin-binding EGF-like growth factor (HB-EGF) and Amphiregulin (AREG) could function as potential tumor markers for ovarian cancer. In this study a set of 231 sera consisting of 131 ovarian cancer patients and 100 healthy age-matched controls were assembled. Nectin-4, HB-EGF and AREG levels of preoperatively collected sera were determined by enzyme-linked immunosorbent assay (ELISA). Our analysis revealed that Nectin-4 and HB-EGF were significantly increased compared to the age-matched control group (*p* < 0.0001, *p* = 0.016). Strikingly, significantly higher Nectin-4 and HB-EGF levels were detected in early-stage FIGO I/II (*p* <0.001; *p* = 0.025) compared to healthy controls. Eighty-four percent (16/19) of patients with low Ca-125 levels showed increased Nectin-4 levels. Our study proposes Nectin-4 and HB-EGF as promising blood-based biomarkers for the detection of early stages of ovarian cancer patients that would not have been detected by Ca-125.

## 1. Introduction

Ovarian cancer (OvCa) is the third most common gynecological malignancy in women and has the highest mortality [1]. In particular, unspecific symptoms and the lack of sufficient screening lead to a diagnosis at an advanced stage of disease in almost two-thirds of all cases [2]. The second challenge of ovarian cancer is the emerging resistance to the applied therapeutic agents, which frequently causes the recurrence of the disease [3]. At present, there is no established screening for the detection of ovarian cancer.

The prognosis of ovarian cancer is largely determined by the tumor stage at initial diagnosis and the success of tumor debulking during surgery [4]. Despite radical surgical procedures and the introduction of new therapeutics such as PARPi in primary therapy, the prognosis of the majority of patients remains poor [5]. In the early stages, the likelihood of recurrence is 20–30%, but rises to 50–75% in the advanced tumor stages. Approximately 50% of all patients relapse within 12 months after initial diagnosis [6]. Therefore, it is of great clinical interest to establish biomarkers that provide insight into treatment response.

Furthermore, effective biomarkers detecting the early stages would significantly improve the prognosis of ovarian cancer. Given that a guideline-compliant therapy is carried out, up to 90% of the early stages can be cured [7]. Nevertheless, only 30% of carcinomas are diagnosed at this stage. Previous attempts to establish a valid screening failed due to the low sensitivity of biomarkers in the early stage of disease (40–50%) [8]. Moreover, the low prevalence of ovarian cancer makes high specificity of biomarkers a prerequisite to achieve a utilizable positive predictive value for clinical practice.

Up to date, there are only a few well-established biomarkers, which have been previously applied for disease monitoring in ovarian cancer. Ca-125 represents the most significant tumor marker for ovarian cancer [9]. However, it does not have sufficient sensitivity or specificity to be used as a screening marker for ovarian cancer. Ca-125 is not organ-specific and may also be detectable in other tumor entities, such as endometrial carcinoma or certain pancreatic tumors [10]. In addition, elevated Ca-125 can be found in approximately 1% of healthy women during menstruation, pregnancy or in endometriosis [11].

A new approach to increasing the predictive power of individual biomarkers is to combine several markers into one score. Thus, in the Risk of Ovarian Malignancy Algorithm (ROMA-Index), which was approved by the Food and Drug Administration (FDA) in 2011, Ca-125 was combined with HE4 to enable better differentiation between benign and malignant diseases [12]. Considering the menopausal status, an estimation of malignancy risk is possible. Despite this progress, early diagnosis remains challenging due to the low detection rate in the early stages [13].

In a previous study, we already proposed a disintegrin and metalloprotease 17 (ADAM17) as a tumor marker in the context of ovarian cancer and demonstrated its importance in the detection of early stages [14]. ADAM17 proteolytically cleaves a substantial number of substrates and plays an important role in the physiological tissue development of organs and tissue regeneration [15]. Overexpression or enhanced activation of ADAM17 in tumor cells has been mostly associated with cancer initiation and progression [16]. Elevated ADAM17 expressions have been shown in most solid tumors, including ovarian cancer [17,18].

This study focuses on three substrates of ADAM17: Nectin-4, heparin-binding epidermal growth factor-like factor (HB-EGF) and Amphiregulin (AREG). All three ligands play critical roles in Epidermal Growth Factor Receptor (EGFR) signaling [19].

Nectin-4 belongs to the family of adhesion molecules that mediate cell–cell interactions and are involved in the formation and stabilization of cell adhesions [20]. In addition, Nectin-4 is able to bind with the extracellular domain growth factors and influence proliferation, invasion, migration and apoptosis processes [21,22]. Nectin-4 has been detected in bronchial, esophageal, gastric, pancreatic, urothelial, breast and ovarian cancer. Elevated expression is linked to a poor prognosis and increased metastatic burden [23,24,25,26]. Nectin-4 is enzymatically cleaved by the proteases ADAM10 and ADAM17 and is detectable in serum [27]. ADAM10 mediates constitutive shedding, whereas increased ADAM17 activity promotes induced shedding. It was shown that Nectin-4 is detectable in the serum of ovarian cancer patients and allows the distinction between benign and malignant ovarian neoplasms [28,29]. Other factors, such as overall survival, progression-free survival or clinical pathological parameters, have only been partially elucidated.

HB-EGF is a transmembrane protein and belongs to the Epidermal Growth Factors (EGF) [30]. It has been implicated in various physiological and pathophysiological processes, such as wound healing and organ development of the heart and the lung, but also cancer initiation [31,32,33]. Among EGFR ligands, it is essentially responsible for tumor growth [34]. Increased expressions have been detected in hepatocellular carcinoma, gastric cancer and breast cancer [35,36]. For bladder cancer, HB-EGF was associated with reduced overall survival [37]. Miyata et al. have already shown that HB-EGF is detectable in the serum of ovarian cancer patients and provides information about prognosis and tumor stage [38].

AREG is physiologically involved in inflammatory processes, tissue regeneration and inflammation [39]. Pathophysiologically, it plays a crucial role in chronic inflammation and in the progression and metastasis of carcinomas [40,41]. AREG is only cleaved by ADAM17 [42]. Several studies have demonstrated its potential as a biomarker due to its role in proliferation and cell survival. For a variety of tumor entities, such as colorectal carcinoma, breast cancer or non-small lung cancer, its prognostic properties as a tumor marker have already been demonstrated [43,44,45]. Since AREG has not been previously studied as a biomarker for ovarian cancer, its predictive and prognostic potential were investigated in this publication.

Even though the EGF ligands Nectin-4, HB-EGF and AREG have been described as rational targets for blood-based detection of ovarian cancer, their application as biomarkers for early-stage ovarian cancer so far has not been shown. The objective of the present study is to investigate the clinical relevance of substrate levels in serum with regard to their predictive and prognostic significance in the context of primary diagnosis of ovarian cancer.

## 2. Materials and Methods

### 2.1. Ethic Statement

The collection and processing of all blood samples was carried out in accordance with the General Data Protection Regulation (GDPR). The ethics application (AZ: B327/10) according to the Declaration of Helsinki and the written consent forms of the patients were obtained in all cases.

### 2.2. Patient Description

To establish Nectin-4, HB-EGF and AREG as potential biomarkers, a patient cohort with histologically confirmed epithelial ovarian cancer was assembled. Initially, 99 patients were studied for AREG and 106 patients for HB-EGF. In order to investigate Nectin-4 more thoroughly, we increased the number of patients in this cohort to 131, all diagnosed between 1998 and 2019. All patients underwent initial tumor debulking surgery. The carcinoma was classified according to the Fédération Internationale de Gynécologie et d’Obstétrique (FIGO) classification and the WHO classification of malignant gynecological tumors. The classification of the FIGO stage was based on a comprehensive consideration of the pathological and clinical aspects. In order to assess the diagnostic significance of the individual biomarkers, the patients’ medical histories were compiled and the following criteria were collected: age at diagnosis, FIGO stage, resection of the tumor (R0 vs. R1) by surgery, histology of the tumor tissue, tumor grading, response to chemotherapy, overall survival (OS) and progression-free survival (PFS) (Table 1). The final update of the PFS and OS was performed in August 2022 with a median follow-up of 46.6 months.

### 2.3. Control Cohort

The healthy controls consisted of 100 female individuals, with no history of either benign or malignant oncological disease. In order to control for potential age effects, controls were between the ages of 50 and 70 years and frequency matched in 10-year age groups to OvCa patient cohort. Particular care was taken to ensure that no chronic diseases such as psoriasis, ulcerative colitis, Crohn’s disease or autoimmune diseases such as rheumatoid arthritis were present. The serum samples were provided by the PopGen 2.0 Biobank Network of the Institute of Epidemiologie in Kiel [46]. These samples were also acquired on the basis of an approved ethics application, in accordance with the Declaration of Helsinki.

### 2.4. Sera Samples

The collection of the serum samples was carried out as described recently [14]. The serum samples were taken as part of routine diagnostics in the Clinic for Gynaecology and Obstetrics at UKSH Campus Kiel. A standard operation procedure was designed for the processing and storage of all samples. After blood withdrawal with a 7.5 mL S-Monovette^®^ (Sarstedt AG & Co., Nuembrecht, Germany), the serum sample was processed within two hours. The samples were centrifuged at 1107× *g* for ten minutes and stored at −80 degrees. Unnecessary freeze–thaw cycles were avoided. Documentation and storage were performed, using the laboratory’s internal database.

### 2.5. Detection of HB-EGF, Nectin-4 and AREG

For detecting HB-EGF, Nectin-4 and AREG in sera, an Enzyme-linked Immunosorbent Assay (ELISA) was used. The corresponding human Duoset Sandwich Elisa (Nectin-4: R&D Systems, #DY2659/AREG: R&D Systems, #DY262/HB-EGF: R&D Systems, #DY259B, Minneapolis, MA, USA) ) was performed according to the modified test instructions. Testing was conducted in a 96-well NUNC-IMMUNO plate (Lot.: 171376, Thermo Fisher Scientific, Roskilde, Denmark). Each sample was measured twice (technical replicates). The optical density (OD) was determined at 450 nm with a laboratory plate reader (Infinite 200, Tecan, Männedorf, Switzerland). For wavelength correction and to avoid measurement inaccuracies, the wavelength at 570 nm was also measured and subtracted from the measured values at 450 nm. The OD was converted into the corresponding concentrations of the respective substrate using Microsoft Excel.

### 2.6. Statistics

All collected patient data were initially encrypted, pseudonymized and then assessed with the statistics program GraphPad PRISM 9; *p*-values < 0.05 are considered statistically significant, 95% confidence intervals are provided. Distributions of serum biomarker levels in different groups are shown as boxplots on a logarithmic scale. As serum biomarker levels showed strongly skewed distributions within groups, nonparametric statistical tests are used, in particular Mann–Whitney test to compare two independent groups and Kruskal–Wallis test for three or more groups, with Dunn’s post hoc test for pairwise comparisons. To assess the diagnostic potential of serum biomarkers to distinguish OvCa patients from healthy controls, a receiver operating characteristic (ROC) curve analysis was performed, including area under the curve (AUC). Sensitivity and specificity for specific cutoff values were determined. Kaplan–Meyer curves were used to illustrate the prognostic relevance of Nectin-4, categorized into quartiles (among patients with follow-up data), regarding PFS and OS. Univariable and multivariable Cox proportional hazard models were fitted and hazard ratios with 95% CI are given for the hazard associated with adjacent quartiles.

## 3. Results

### 3.1. Nectin-4 and HB-EGF Levels Are Elevated in Ovarian Cancer Patients

**Figure 1:**
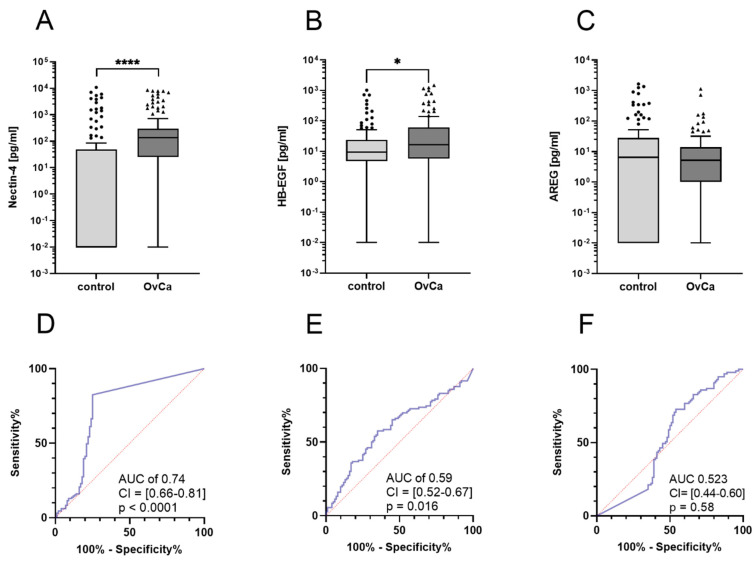
Boxplots of Nectin-4, HB-EGF and AREG levels at primary diagnosis. (**A**): Nectin-4 is elevated in OvCa patients, Mann–Whitney test **** (*p* < 0.0001). (**B**): HB-EGF is increased in OvCa patients, Mann–Whitney test * (*p* = 0.016). (**C**): AREG levels of the patient cohort and the control group were similar, Mann–Whitney test (*p* = 0.57). (**D**): Receiver operating characteristic (ROC) of Nectin-4. (**E**): ROC curve of HB-EGF. (**F**): ROC curve of AREG.

To investigate whether the ADAM17 substrates Nectin-4, HB-EGF and AREG could potentially be used as diagnostic tumor markers for ovarian cancer, a cohort of 131 patients with primary ovarian cancer was assembled and characterized with respect to their clinicopathologic parameters. In addition, a control cohort of 100 age-matched female donors was compiled who had no history of oncologic disease. Ovarian cancer patients showed significantly higher concentrations of both Nectin-4 (median: OvCa patients: 137.7 pg/mL, control: 0 pg/mL, *p* < 0.0001) and HB-EGF (median: OvCa patients: 16.4 pg/mL, control: 9.4 pg/mL, *p* = 0.016) compared to the control cohort (Figure 1A,B). No significant difference was shown for AREG levels between both groups (median: OvCa patients: 5.2 pg/mL, control: 6.5 pg/mL, *p* = 0.57) (Figure 1C). ROC curve analysis shows the diagnostic potential of each biomarker, with an AUC of 0.74 (95% CI = [0.66–0.81], *p* < 0.0001) for Nectin-4 and an AUC of 0.59 (95%CI = [0.52–0.67], *p* = 0.016) for HB-EGF. For Nectin-4, a sensitivity of 82.4% and a specificity of 75.0% would be reached at a cutoff value of 2.672 pg/mL. For HB-EGF, a cutoff value of 1.70 pg/mL would yield a sensitivity of 90% and a specificity of 9%. These results demonstrate that two of the three substrates could be useful diagnostic biomarkers for ovarian cancer.

Since we showed a significant increase in Nectin-4 and HB-EGF in ovarian cancer compared to the control group, these two were further investigated in this study. In the following, we examine whether the substrate levels also correlate with the clinical parameters obtained, such as FIGO stage, postoperative resection status or histologic features.

### 3.2. High Nectin-4 Levels Indicate Early-Stage Ovarian Cancer Patients

**Figure 2:**
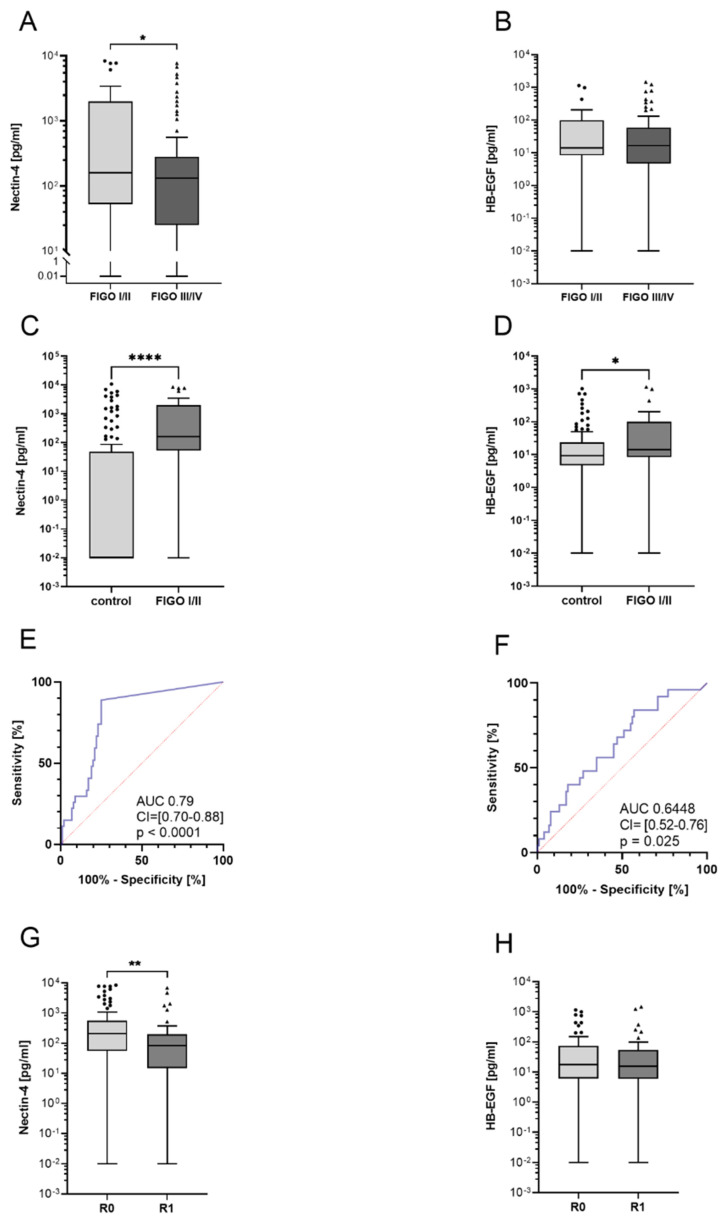
(**A**): Significantly higher concentrations of Nectin-4 in FIGO I/II (26 cases) compared to FIGO III/IV (104 cases), Mann–Whitney test * (*p* = 0.043). (**B**): No significant difference in HB-EGF levels between early and advanced FIGO stages, Mann–Whitney test (*p* = 0.59). (**C**): Nectin-4 levels were significantly increased in FIGO I/II stages (26 cases) vs. 100 healthy controls, Mann–Whitney test **** (*p* < 0.0001). (**D**): Significantly elevated HB-EGF levels between control group vs. early FIGO I/II stages, Mann–Whitney test * (*p* = 0.025). (**E**): Receiver operating characteristic (ROC) of Nectin-4. (**F**): ROC curve of HB-EGF. (**G**): Significantly elevated Nectin-4 levels in 72 patients without macroscopic tumor remnant (R0) compared to 56 patients with macroscopic tumor remnant (R1), Mann–Whitney test ** (*p* = 0.0021). (**H**): No increased HB-EGF levels between R0 and R1. Mann–Whitney test (*p* = 0.74). All panels except for E and F show boxplots.

Early diagnosis is crucial for the overall prognosis of ovarian cancer and has a decisive impact on further therapy. Therefore, it was investigated whether Nectin-4 or HB-EGF differ between FIGO stages. We compared the early stages I and II with the prognostically unfavorable advanced stages III and IV. Interestingly, Nectin-4 levels in early stages FIGO I/II were 44% higher compared to FIGO III/IV (Mann–Whitney test, *p* = 0.043) (Figure 2A). ROC analysis shows an AUC of 0.63 (CI = [0.50–0.75], *p* = 0.04), allowing a very clear differentiation between early- and late-stage OvCa patients (FIGO I/II vs. FIGO III/IV). At a cutoff value of 340.4 pg/mL, Nectin-4 had a sensitivity of 82.7% and a specificity of 42.3% to distinguish between stages. There was no significant difference in HB-EGF serum levels between FIGO stages (Mann–Whitney test, *p* = 0.59) (Figure 2B).

In order to investigate whether the early stages can be separated from healthy controls by Nectin-4 and HB-EGF, the substrate levels during the early stages were compared with those of the control group. The comparison showed significantly increased sera levels for both Nectin-4 (Mann–Whitney test, *p* < 0.001) and HB-EGF (Mann–Whitney test, *p* = 0.025) in the early stages compared to healthy controls (Figure 2C,D). To determine suitable Nectin-4 cutoff values, we performed ROC analyses that showed an AUC of 0.79 (95%CI = [0.70–0.88], *p* < 0.0001) for Nectin-4. With a Nectin-4 cutoff value of 12.26 pg/mL, the sensitivity was 88.5% and specificity was 75% to distinguish between early FIGO stages I/II and healthy controls. HB-EGF had a lower diagnostic potential with an AUC of 0.64 (95%CI = [0.52–0.76], *p* = 0.025). At a HB-EGF cutoff of 5.42 pg/mL, a sensitivity of 92% and a specificity of 29% were obtained (Figure 2E,F). This underlines the diagnostic value of Nectin-4 and HB-EGF for the detection of early stages of ovarian cancer.

Complete tumor debulking under surgery has a critical impact on the overall prognosis. Therefore, we compared the substrate levels of patients with complete tumor resection (R0) to those with a macroscopic remaining tumor (R1). Significantly increased Nectin-4 levels (Mann–Whitney test, *p* = 0.0021) are shown in patients with a macroscopic complete resection (Figure 2G). This is congruent with the above-mentioned findings, as complete resection is more likely to be achieved in the early stages. No difference was demonstrated for HB-EGF (Mann–Whitney test, *p* = 0.74) with respect to tumor resection (Figure 2H). This underscores that Nectin-4 is the more reliable tumor marker.

### 3.3. No Differences in Nectin-4 or HB-EGF Levels in Histological Subtypes or Tumor Grading

**Figure 3:**
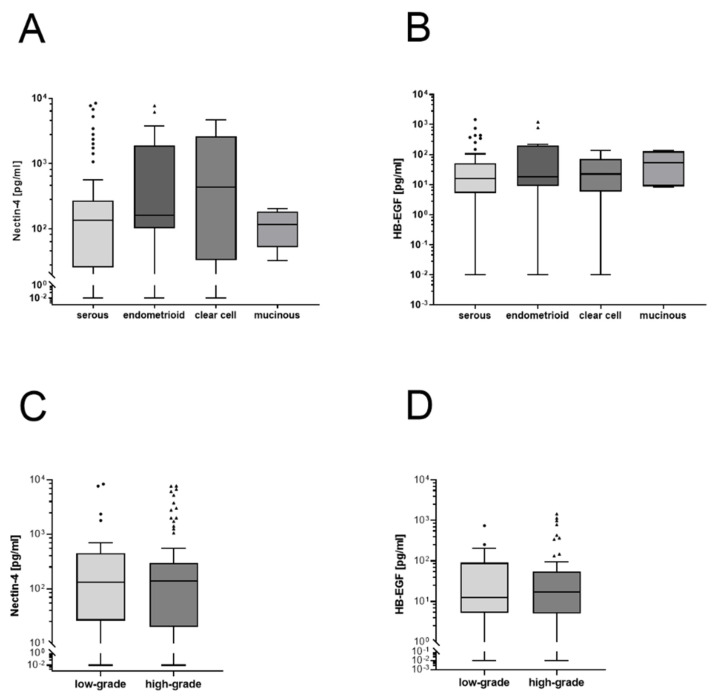
Boxplots of Nectin-4 and HB-EGF levels in serum according to histological subtypes and grading. (**A**): No significant difference between endometrioid, serous, clear cell and mucinous subtypes regarding the Nectin-4 levels, Kruskal–Wallis test (*p* = 0.127). (**B**): No significant differences in HB-EGF levels between histological subtypes, Kruskal–Wallis test (*p* = 0.73). (**C**): No evidence of differences in Nectin-4 level with grading, Mann–Whitney test (*p* = 0.77). (**D**): No evidence of differences in HB-EGF level with grading, Mann–Whitney test (*p* = 0.66).

To investigate differences in substrate levels between histological entities, we compared serous, endometrioid, clear cell and mucinous histological subtypes. No significant difference was shown between histological subtypes for either Nectin-4 or HB-EGF. As a second pathological parameter, tumor grading was investigated. There was no difference between the low-grade and high-grade carcinomas in both Nectin-4 and HB-EGF serum levels (Figure 3C,D).

### 3.4. Ovarian Cancer Diagnostics When Ca-125 Fails

**Figure 4:**
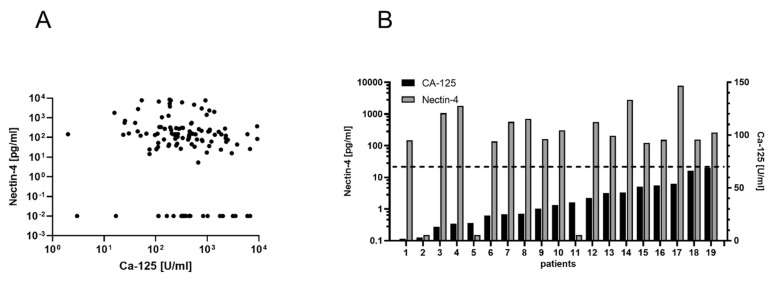
(**A**) Scatterplot of Nectin-4 and Ca-125 in 122 OvCa patients. (**B**) Most patients with low Ca-125 levels have high Nectin-4 levels. Gray columns show Nectin-4 levels and black columns show Ca-125 levels of individual patients. The dashed horizontal line indicates the cutoff value for Ca-125 of 70 U/mL.

To investigate whether Ca-125 and Nectin-4 could complement each other in the diagnosis of ovarian cancer, we investigated subgroups of patients with particularly low values of Ca-125. In our cohort, both Ca-125 and Nectin-4 were determined before surgery in 122/131 patients. Among the 19 patients (15%) with the lowest Ca-125 levels (<70 U/mL) in our sample, the majority (16/19 = 84%) had Nectin-4 levels above 12.26 pg/mL (median: 283.9 pg/mL) and 7 (37%) of these patients were in an early stage of the disease (Figure 4). On the other hand, of those 23 patients with low levels of Nectin-4 (below the detection limit of our assay), only 3 (13%) had Ca-125 levels below 70 U/mL. Furthermore, all patients who would have remained undetected by both Ca-125 and Nectin-4 (low values in both) had elevated HB-EGF concentrations (over 11 pg/mL). These results indicate that Nectin-4 and HB-EGF have great potential to complement Ca-125 in the diagnosis of ovarian cancer.

### 3.5. Prognostic Relevance of ADAM17 Substrates

**Figure 5:**
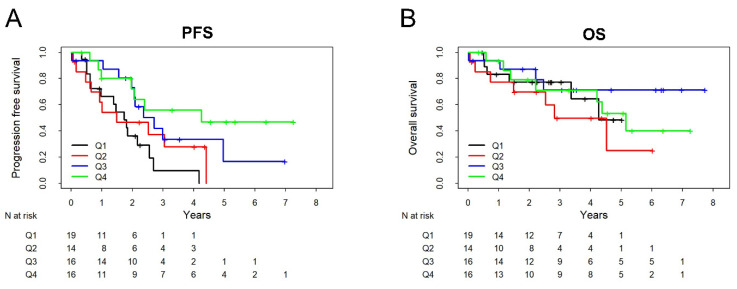
Prognostic relevance of Nectin-4 at primary diagnosis for PFS (**A**) and OS (**B**). Kaplan–Meier curves for patients with Nectin-4 levels categorized in quartiles (Q1–Q4).

To investigate the prognostic relevance of Nectin-4 regarding PFS and OS, those patients with available follow-up data (PFS: *n* = 65/OS: *n* = 65) were divided into quartiles according to their serum substrate levels. Kaplan–Meier curves and univariable Cox proportional hazards analysis were used to analyze the prognostic significance of each substrate at primary diagnosis. Lower Nectin-4 levels were associated with significantly reduced PFS (HR 0.64 [95% Cl 0.48 to 0.85] *p* = 0.002) but not with significantly reduced OS (HR 0.93 [95% Cl 0.65 to 1.32] *p* = 0.7; Figure 5). In a multivariable Cox proportional hazards model, adjusted for FIGO stage, histological subtype and resection (R0 vs. R1), Nectin-4 level (categorized in quartiles) was still a significant predictor of PFS (HR 0.67 [95% CI 0.49 to 0.92]), *p* = 0.012). In a model which additionally includes Ca-125, Nectin-4 remained significant (*p* = 0.04) while Ca-125 did not provide additional prognostic value (*p* = 0.56) for PFS beyond FIGO stage, histological subtype and resection.

## 4. Discussion

Owing to the lack of early symptoms, the majority of ovarian cancers are diagnosed at an advanced stage of the disease. While the cure rate in FIGO stage I is still over 90%, the 5-year overall survival in FIGO stage IV is less than 20% [47]. In order to improve prognosis, it is of great importance to establish reliable early detection methods to diagnose and treat ovarian cancer at an early stage of the disease. Nevertheless, blood-based early diagnosis is often proving difficult because many tumor markers only increase during tumor progression and are tumor mass dependent [48].

For the diagnosis of malignant diseases, many attempts have been made to establish alternatives to tissue biopsy, which in many cases needs invasive procedures. Noninvasive approaches try to detect components released by the tumor into blood, ascites, saliva or urine. For both oral cancer and bladder cancer, detection of biomarkers in saliva or urine has been shown to be a promising adjunct to clinical examination and imaging [49,50]. For other tumor entities, such as colon or ovarian cancer, the establishment of liquid-based biomarkers is more difficult because they are less specific. Ovarian cancer carcinogenesis is often associated with chronic inflammatory processes. Therefore, Perez et al. demonstrated that the inflammatory chemokine C-X-C motif chemokine ligand 13 (CXCL13) is associated with increased ovarian cancer risk [51]. However, inflammatory chemokines are also increasingly expressed in other tumor entities, such as lung carcinoma or lymphoma [52,53]. Therefore, next to detection of various glycoproteins, such as Ca-125 or HE4, there are innovative approaches, such as the detection of circulating tumor DNA, or extracellular vesicles in serum [54,55]. Moreover, in the future, a combination of different markers and/or different strategies might be the key for early detection of OvCa. Therefore, in the present study, we evaluate the ADAM17 substrates Nectin-4, HB-EGF and AREG as a potential blood-based detection method for ovarian cancer.

It was shown that Nectin-4, HB-EGF, and AREG are proteolytically cleaved by the metalloprotease ADAM17 and represent essential factors in the regulation of ovarian cancer. ADAM17 is a key mediator of several downstream survival pathways such as phosphoinositide 3-kinase and serine/threonine kinase-AKT (PI3K/AKT), epidermal growth factor receptor activation (EGFR) and mitogen-activated protein kinase (MAPK) [56]. For both breast and ovarian carcinomas, it has been shown that the increased release of various substrates such as AREG, transforming growth factor alpha (TGF-alpha), and HB-EGF activates the EGFR receptor and contributes to tumorigenesis and disease progression [57,58,59]. We have previously shown that ADAM17 is elevated in the blood of ovarian cancer patients. Interestingly, early-stage FIGO I/II had significantly elevated ADAM17 levels compared to healthy controls. [14,60]. To gain insight into the functional activity of ADAM17, we selected Nectin-4, HB-EGF and AREG as surrogate markers for ADAM17 activity and evaluated them as potential tumor markers for ovarian cancer.

In the past, there were a series of attempts to establish AREG as a tumor marker for different tumor entities, such as breast, lung, hepatocellular or pancreatic carcinoma [43,61,62,63,64,65]. While several studies have been successful in predicting treatment response, such as to cetuximab in colorectal carcinoma, the establishment of a screening marker has only been possible for hepatocellular carcinoma [62,64]. Peterson et al., evaluating AREG as a screening marker for breast carcinoma, could not demonstrate a difference between the healthy and diseased cohorts [65]. Interestingly, Ishikawa et al. found significantly higher AREG levels in men with nonsmall cell lung cancer compared with women [63]. Moreover, AREG is not tumor-specific, but also occurs in the serum of other diseases, such as graft-versus-host disease, idiopathic inflammatory myopathy and asthma [66,67,68]. It is known that AREG plays an essential role in the regulation of estrogen balance and can be induced in a progesterone-dependent manner [69,70]. Consequently, Kjær et al. detected differential AREG concentrations of 0.5–50 pg/mL in healthy women aged 26–78 years [71]. In our study, AREG was detected in the serum of patients with ovarian cancer, but there was no significant difference compared to the control group. This suggests that AREG has limited value as a tumor marker, so we restricted our subsequent studies to Nectin-4 and HB-EGF.

The predictive and prognostic properties of HB-EGF have been studied in several studies. Initially, Kasai et al. demonstrated in a smaller cohort that HB-EGF was elevated in ovarian cancer patients compared to a healthy control group [72]. Miyata et al. were able to confirm these results in a larger cohort and further proved that increased HB-EGF levels were associated with shorter PFS. However, a correlation between HB-EGF and histological subtype or HB-EGF and postoperative tumor residual could not be verified [38]. These results are in line with our findings. Furthermore, our data demonstrate a slight difference in HB-EGF levels between the early stages and healthy controls. This suggests that HB-EGF may contribute to the early detection of ovarian cancer. Accordingly, this suggests increased ADAM17 activity in the early stages.

In this study, Nectin-4 has been most intensively investigated as a potential blood-based tumor marker for ovarian cancer. Overexpression of Nectin-4 has already been shown for a variety of tumor entities [23,24,25]. Nectin-4 plays a crucial role in tumor cell aggregation, migration and tumor progression because it can lead to the formation of cancer spheroids by increasing cell adhesion [26]. DeRycke et al. previously demonstrated that Nectin-4 is elevated in tissue samples and is detectable in the blood of ovarian cancer. In addition, they showed that Nectin-4 enables differentiation between benign gynecological diseases and ovarian cancer [29]. In contrast to DeRycke, who did not provide FIGO-stage information, we were able to show that detection of early stages is possible. Furthermore, we included an age-matched healthy donor control. Significantly elevated Nectin-4 levels were detected in the blood of patients with ovarian cancer. At a cutoff value of 2.672 pg/mL, sensitivity was 82.4% and specificity was 75%. Thus, our values are considerably higher than those of DeRycke et al., who report a sensitivity of 53% and a specificity of 77% for a cutoff value of 0 pg/mL [29]. Nabih et al. set the cutoff at 11.7 pg/mL and calculated a sensitivity of 84.7% and a specificity of 95.7% [28]. Different cohort sizes and histologic subgroup distributions may be responsible for the variability in reported cutoff values. Although this does not warrant sole use as a screening marker, combination with other tumor markers is conceivable.

A decisive way to improve overall survival in ovarian cancer is to establish the initial diagnosis in the early stages. Since Nabih et al. were already able to show an association between Nectin-4 and FIGO stage in 39 ovarian cancer patients, we wanted to confirm this in a larger cohort [28]. Therefore, we analyzed Nectin-4 concentrations according to FIGO stages in *n* = 131 cases. Interestingly, the levels in the early stages were more than 40% higher than in the prognostically unfavorable advanced stages. In addition, distinction between the early stages and the control group was significantly improved at a cutoff value of 12.26 pg/mL with a sensitivity of 88.5%. Our data suggest that Nectin-4 is rather a surrogate marker for initiation and progression of early ovarian cancer than a measurement of tumor mass such as Ca-125. This could be due to an ADAM17-dependent release of Nectin-4 and HB-EGF [27,73], as we recently showed [74]. Our finding is further supported by notion that ADAM17 mediates the invasive growth of the carcinoma [75]. Moreover, it is necessary to consider not only the role of the tumor but also the tumor microenvironment (TME) in carcinogenesis [76]. It consists of a variety of cells such as endothelial cells, cancer-associated fibroblasts (CAF), immune cells and stem cells [77,78]. The interplay of these cells releases growth factors from the surrounding stroma and establishes paracrine signaling cascades [79]. A key regulator in tumor metabolism, promotion of uncontrolled tumor cell growth, and autogenously cell interaction by releasing growth factors and overexpressing corresponding receptors is the metalloprotease ADAM17, of which is reported to cleave a substantial number of substrates [80,81]. Since Nectin-4 seems to be independent of tumor mass, it is at least partly expressed by tumor-surrounding tissues. Therefore an increase in biomarker levels would already be detectable in the early stages [75].

One of the major prognostic factors of ovarian cancer is the postoperative residual tumor burden. DuBois et al. showed that complete resection significantly improves both PFS and OS [82]. Our study revealed that patients with complete resection had significantly increased Nectin-4 levels preoperatively. In line with these findings, Nectin-4 levels are measurably elevated in both early stages and complete resection. This is understandable given that macroscopically tumor-free resection can be achieved much more frequently in the early stages. The analysis of our cohort confirms that in 95% of early stages, complete resection can be successfully performed. Considering that increased Nectin-4 levels are associated with improved surgical outcome, patients with a more favorable risk profile can be identified.

Owing to the limited sensitivity and specificity of Ca-125, there have been many efforts to close this diagnostic gap. Therefore, several algorithms were established that included multiple tumor markers in their analysis. For example, the ROMA index, a combination of Ca-125 and HE4, was able to achieve a sensitivity of 90% and a specificity of 91% [83]. Our study revealed that Nectin-4 and Ca-125 may provide complementary diagnostic information. Thus, we focused on those patients with the lowest Ca-125 levels in our patient cohort. Interestingly, >80% of patients within this group had significantly elevated Nectin-4 levels and over one-third of these patients were in an early stage of the disease. This opens the possibility of identifying patients who would not have been detected by Ca-125. The particular strength of Nectin-4 seems to be the diagnosis of early stages. Here, an AUC of 0.79 could be calculated. Tcherkassova et al. reported the AUC of Ca-125 for differentiation between healthy donors and FIGO I/II as 0.80 [5]. Mukama et al. analyzed 92 biomarkers for the detection of early ovarian cancer. Only nine markers had an AUC above 0.70. Ca-125 and HE4 again showed the highest discrimination with an AUC of 0.77 and 0.73, respectively [6]. This indicates that Nectin-4 and Ca-125 are quite comparable in their diagnostic potential for early stages and as we can show in our study they might be complementary in the sense that most patients with low Ca-125 had high Nectin-4 level and vice versa. Overall, we are aware that Nectin-4 alone cannot be used in the diagnosis of ovarian cancer, but a combination in a biomarker panel should be aimed for. Finally, it has to be stressed that serum biomarkers might be used for inexpensive and low-invasive screening of unsymptomatic women but would never be used to establish a definite diagnosis; a positive screening test result would always need to be confirmed by gold-standard diagnostics. For use as a screening test, high sensitivity is most important and this could be achieved by a combination of serum biomarkers.

In comparison to the predictive qualities, we also investigated the prognostic potential of Nectin-4, showing that preoperatively increased Nectin-4 levels were associated with a significantly prolonged PFS. No difference was observed with respect to OS. This is in line with our previous findings that increased Nectin-4 levels correlate with the prognostically more favorable early stages and complete resection. Whether Nectin-4 is related to PFS or OS is evaluated differently in the literature. DeRycke et al. could not show a correlation with either PFS or OS based on the expression of Nectin-4 in tumor tissue [29]. Bekos et al. showed only for high-grade serous late-stage ovarian cancer (FIGO III/IV) that an increased expression in tumor tissue is associated with a reduced OS [84]. Buchanan et al. also examined gene expression of Nectin-4 only for advanced FIGO stages III/IV and showed that increased Nectin-4 expression was associated with reduced PFS [27]. Early stages FIGO I/II were not considered in these studies. Therefore, our study is the first to provide a correlation between PFS and OS based on serum protein levels, including early stages of ovarian cancer.

In recent decades, multidisciplinary approaches have steadily improved the prognosis of ovarian cancer patients [85]. The use of novel molecular and clinical investigations has been crucial in improving the diagnosis and treatment of ovarian cancer. In particular, proteogenomic profiling has allowed the identification of new targets that constitute potential biomarkers [86]. A promising approach is the combination of different serological biomarkers that complement each other in their predictive and prognostic properties [87]. Of particular importance are panels of biomarkers that detect patients without an elevated Ca-125 already in the early stages [88]. The establishment of an effective screening system that allows detection in the early stages could significantly reduce the mortality of ovarian cancer. In this study, we propose Nectin-4 as a potential biomarker for ovarian cancer that has high potential to improve the detection of ovarian cancer as part of a biomarker panel, in particular by identifying early stages of the disease.

## 5. Conclusions

The ADAM17 substrates Nectin-4 and HB-EGF appear to be promising markers for the detection of early stages of ovarian cancer and are associated with prognostically favorable parameters, such as early FIGO stage and successful tumor debulking. It is noteworthy that Nectin-4, complementary to Ca-125, allows the identification of patients who would otherwise have remained falsely undetected. Owing to their easy detectability in the blood, they could be implemented in the diagnostic screening for ovarian cancer and consequently improve the therapy options for the patients as well as the individual prognosis.

## Figures and Tables

**Table 1:** Clinical parameters of the patient cohort. Shown in the table: Histology of tumor, tumor grading, FIGO stage, resection of tumor mass under surgery, response to chemotherapy, overall survival (OS), progression-free survival (PFS) and age. Not applicable (n.a.).

Clinical Parameter	Characteristics	N	Percentage
Histology	Serous	97	74.0%
	Endometrioid	17	13.0%
	Clear-cell	8	6.1%
	Mucinous	4	3.0%
	Others	5	3.8%
Grading	Low	29	22.1%
	High	90	68.7%
	n.a.	12	9.1%
FIGO	I	20	15.3%
	II	6	4.5%
	III	81	61.8%
	IV	23	17.5%
	n.a.	1	0.9%
Resection of the tumor	R0	72	54.9%
	R1	56	42.7%
	n.a.	3	2.3%
Response to chemotherapy	Sensitive	86	65.6%
	Resistant	24	18.3%
	n.a.	21	16.0%
Progression-free survival	Median 23.0/Min.: 0/Max.: 87/25.P.: 10.5/75.P.: 35.5 (in month)		
Overall survival	Median 33.8/Min.: 0.2/Max.: 94/25.P.: 15.7/75.P.: 55.7 (in month)		
Age	Mean 60.3 +/− 10.4 /Min.: 29/Max.: 89 (in years)		

## Data Availability

The data presented in this study can be obtained from the corresponding Author.
